# ATPIF1-driven metabolic remodeling in osteoclasts promotes age-related cortical bone loss

**DOI:** 10.21203/rs.3.rs-11273064/v1

**Published:** 2026-10-29

**Authors:** Ha-Neui Kim, Gareeballah Adam, Ankita Chalke, Aaron Warren, Kimberly Richardson, Adriana Marques-Carvalho, Mikesha Carter, Hanwen Fan, Yuxiao Zhou, Jeff Thostenson, Farah Alturkmani, Benjamin Stronach, Jeffrey Stambough, Elena Ambrogini, Se-Ran Jun, Maria Almeida

**Affiliations:** University of Arkansas for Medical Sciences; University of Arkansas for Medical Sciences; University of Arkansas for Medical Sciences; University of Arkansas for Medical Sciences; University of Arkansas for Medical Sciences; University of Arkansas for Medical Sciences; University of Arkansas for Medical Sciences; Texas A&M University; Texas A&M University; University of Arkansas for Medical Sciences; University of Arkansas for Medical Sciences; University of Arkansas for Medical Sciences; University of Arkansas for Medical Sciences; University of Arkansas for Medical Sciences; University of Arkansas for Medical Sciences; University of Arkansas for Medical Sciences

## Abstract

Age-related cortical bone loss is driven in part by increased osteoclast activity, but the mechanisms that sustain osteoclast function during skeletal aging remain poorly defined. Here, we show that aging remodels osteoclast metabolism, increasing glycolytic ATP production and reshaping metabolites linked to early tricarboxylic acid (TCA) cycle metabolism. Metabolomic profiling identified a glycolysis-coupled TCA node marked by increased isocitrate and -ketoglutarate in aged osteoclasts. These cells also exhibited prolonged survival and reduced activation of regulated cell-death pathways; in mice, this was accompanied by increased bone resorption, while human osteoclasts from aged donors similarly showed prolonged persistence. Mechanistically, ATPase inhibitory factor 1 (ATPIF1) was required for this aging-associated metabolic state. Osteoclast-lineage deletion of Atpif1 reduced IDH3A expression, -ketoglutarate accumulation, glycolytic ATP production, and resorptive function ex vivo, while attenuating age-related cortical bone loss and porosity in vivo. Pharmacologic perturbation of IDH3-linked metabolism similarly suppressed glycolytic ATP production and osteoclast activity and increased cortical bone mass in aged mice. Together, these findings identify glycolysis-centered metabolic rewiring as a mechanism that sustains osteoclast survival and resorption during skeletal aging and implicate an ATPIF1–IDH3A metabolic axis as a potential therapeutic target for age-associated osteoporosis.

## Introduction

Bone homeostasis is maintained by the balanced actions of bone-resorbing osteoclasts and bone-forming osteoblasts^1,2^. Osteoporosis, the most common metabolic bone disease, is characterized by low bone mass, skeletal fragility and increased fracture risk, and aging is a major driver of this process^3–5^. Age-related fragility arises in large part from progressive thinning and increased porosity of cortical bone, the compact outer layer of bones. These processes are largely driven by increased bone resorption^6,7^. Current therapies can halt bone resorption, but their long-term efficacy remains incomplete and their use can be limited by adverse effects^8,9^. A better understanding of the molecular mechanisms that govern osteoclast activity may therefore inform more effective therapies for osteoporosis.

Osteoclasts differentiate from monocytic precursors in response to macrophage colony-stimulating factor (M-CSF) and receptor activator of nuclear factor-kB ligand (RANKL), and acquire a highly energy-demanding program required for bone resorption^10–12^. During osteoclastogenesis, RANKL promotes mitochondrial biogenesis and respiratory activity, consistent with the energetic demands of proton transport and matrix degradation^11,13^. However, high cellular energy demand can also be supported by increased glycolytic ATP production even in the presence of oxygen, while mitochondrial TCA cycle activity remains engaged. Such coordinated use of glycolysis and mitochondrial metabolism is characteristic of Warburg-like metabolic reprogramming and can support energetic demand, biosynthetic adaptation, and cell survival under conditions of stress or high functional demand^14–16^. Within the TCA cycle, isocitrate dehydrogenase 3 (IDH3) catalyzes the conversion of isocitrate to α-ketoglutarate, a key metabolic step that can influence mitochondrial carbon metabolism and cellular metabolic adaptation^17,18^. Whether osteoclasts engage a similar metabolic program, whether osteoclast metabolism changes with aging, and whether such changes contribute to osteoporosis remain poorly understood.

We have recently shown that the mitochondrial deacetylase SIRT3 in osteoclasts promotes bone resorption and age-related bone loss, with more pronounced effects in female mice and in cortical than trabecular bone^13,19^. Proteomic and acetylomic analyses further revealed that SIRT3 remodels the mitochondrial acetylome and identified ATPase inhibitory factor 1 (ATPIF1), a regulator of mitochondrial quality control and bioenergetics, as a prominent downstream target^19^. Nonetheless, the metabolic program elicited by ATPIF1 in osteoclast mitochondria and its relevance to age-related cortical bone loss remain unresolved.

To define the metabolic basis of osteoclast activation during skeletal aging, we combined metabolomic profiling across osteoclast differentiation stages with *ex vivo* and *in vivo* functional analyses in aged mice. We first identified aging-associated metabolic changes in mature osteoclasts, then tested their functional relevance using osteoclast-lineage deletion of *Atpif1* and pharmacologic perturbation of IDH3-linked metabolism, and finally examined how these interventions influenced cortical bone loss *in vivo*.

In this study, we show that skeletal aging promotes a Warburg-like, glycolysis-coupled TCA metabolic program in mature osteoclasts that prolongs cell survival, suppresses multiple cell death pathways, and increases endocortical bone resorption in female mice. Mechanistically, this program is linked to ATPIF1– IDH3-associated remodeling of an early TCA cycle node marked by increased isocitrate and α-ketoglutarate. Pharmacological inhibition of this pathway attenuated bone resorption and increased cortical bone mass in aged female mice, identifying osteoclast metabolic remodeling as a potential therapeutic target for osteoporosis and skeletal aging.

## MATERIALS AND METHODS

### Sex as a biological variable.

This study focused on female mice because age-related cortical bone loss and the skeletal effects of osteoclast mitochondrial dysfunction are particularly pronounced in females in our experimental models. Human osteoclast studies were likewise performed using bone marrow samples obtained from female donors undergoing hip replacement surgery. Whether the metabolic mechanisms identified here are conserved in males remains to be determined.

### Animals.

Naturally aged female C57BL/6 mice (18–24 months old) were obtained from the National Institute on Aging, and young adult female C57BL/6 mice (4–6 months old) were purchased from The Jackson Laboratory. Mice were housed 3–5 per cage with ad libitum access to food and water in Association for Assessment and Accreditation of Laboratory Animal Care International-accredited animal facilities. Animals with visible tumors or evidence of leukemia were excluded from experiments and analyses. The animal facility was maintained at 20–26°C and 30–70% relative humidity under a 12-h light/12-h dark cycle.

The *Atpif1* conditional allele (Atpif1^tm1c(EUCOMM)Wtsi^; EM:05233) was obtained from the Mary Lyon Centre at MRC Harwell through the European Mouse Mutant Archive and was originally derived from a Wellcome Trust Sanger Institute EUCOMM allele. This post-Flp conditional allele contains loxP sites flanking exon 2 of *Atpif1* and was maintained on a C57BL/6 background.

To achieve myeloid-lineage deletion, *Atpif1*^fl/fl^ mice were crossed with LysM-Cre transgenic mice (The Jackson Laboratory, stock no. 004781). Experimental cohorts were generated by intercrossing *Atpif1*^fl/+^;LysM-Cre^+^ offspring to obtain *Atpif1*^fl/fl^ littermate controls and *Atpif1*^fl/fl^;LysM-Cre^+^ conditional knockout mice (*Atpif1*^ΔLysM^). *Atpif1* genotyping was performed using a forward primer (5′-TGCCTGACATTGGTATTGGG-3′) and a reverse primer (5′-GTGCAGCTTGTGGGAGTCAG-3′), yielding 396-bp wild-type and 605-bp floxed allele products. The mutant-specific allele was detected using the same forward primer together with CAS_R1_Term (5′-TCGTGGTATCGTTATGCGCC-3′), yielding a 114-bp product. To investigate the role of ATPIF1 in age-related bone loss, female *Atpif1*^ΔLysM^ mice and littermate controls were aged to 18 months under standard housing conditions.

For the gossypol study, aged (18-month-old) female C57BL/6 mice were intraperitoneally administered vehicle (3% DMSO in sterile saline) or gossypol (25 mg/kg) three times per week for 12 weeks. At the study endpoint, mice were euthanized, and femurs and serum were collected for analysis.

LysM-Cre;TdTomato mice were generated by intercrossing LysM-Cre mice with Ai9 reporter mice (TdTomato; The Jackson Laboratory; stock no.007909).

### Bone density and microarchitectural analysis.

BMD measurements were performed using DXA with a PIXImus densitometer (GE Lunar) in mice anesthetized with 2% isoflurane. Scans of the entire left femur or lumbar vertebrae (L1–L6) were used for BMD measurements, and data were analyzed as previously described^5^. After euthanasia, left femurs and fifth lumbar vertebrae (L5) were dissected, cleaned of soft tissue, fixed in 10% Millonig’s formalin containing 5% sucrose for 24 h, and gradually dehydrated in 100% ethanol. Bones were scanned at medium resolution (12 μm isotropic voxel size; E = 55 kVp, I = 145 μA, integration time = 200 ms) using a Scanco Medical micro-CT40 scanner and analyzed with Scanco Eval Program v.6.0. Scans were reconstructed into 3D voxel images (1024 × 1024-pixel matrices for each planar stack).

Cortical dimensions at the femoral diaphysis were determined using 18 slices at the midshaft. Cortical thickness at the metaphysis was measured starting 8–10 slices from the growth plate and extending proximally for 151 slices, with cross-sectional contours drawn to exclude trabecular elements. Cortical porosity at the distal metaphysis was determined in an extended cortical region beginning at the same metaphyseal starting point used for thickness measurements and spanning a substantially larger portion of the femur.

The lumbar vertebrae were scanned from the rostral to the caudal growth plate to obtain approximately 230 slices. Contours were drawn around the anterior vertebral body immediately below the superior growth plate, excluding primary spongiosa, cortical regions, and bone outside the vertebral body plate. Trabecular and cortical bone were segmented using grayscale thresholds of 220 and 200 mg/cm^3^, respectively. Trabecular morphometric quantification was performed using a distance-transform method without assumptions regarding rod- or plate-like geometry. Bone nomenclature conforms to the recommendations of the American Society for Bone and Mineral Research^20^.

### In situ mechanical loading and digital volume correlation analysis.

To assess bone mechanical behavior in aged wild-type and *Atpif1*^ΔLysM^ mice, left tibiae were collected from aged mice (n = 4 per group). The distal ends of the tibiae were secured to the lower compression jaw using polymethyl methacrylate (PMMA, Ortho-Jet, Lang Dental Manufacturing, Wheeling, IL). The proximal ends were covered with a thin PMMA layer to establish parallel loading surfaces. *In situ* axial compression testing was performed using a CT5000 loading stage (Deben, Suffolk, UK) integrated into an Xradia 620 Versa micro-CT system (Zeiss, Oberkochen, Germany). For each tibia, baseline micro-CT scan was acquired under no-load conditions, followed by a second scan acquired after the bone reached mechanical stability under a 1.25 N compressive load applied along the longitudinal axis of the bone. All scans were performed at 90 kV and 12 W with an isotropic voxel size of 10.96 mm. Full-field deformation inside the bone was quantified using digital volume correlation analysis of the no-load and loaded micro-CT scan volumes in DaVis software (LaVision GmbH, Göttingen, Germany). A multipass correlation scheme was used, yielding a final correlation window size of 350.7 μm. Spurious displacement vectors, defined by a correlation coefficient < 0.5 or a peak ratio < 2.5, were removed and replaced by local interpolation. Strain fields were derived from displacement gradients and mapped onto the three-dimensional bone microstructure using Avizo software (Thermo Fisher Scientific, Waltham, MA, USA) for visualization of three-dimensional von Mises strain contours. The average von Mises strain was calculated within a 0.3-mm-high volume of interest located 0.6 mm below the growth plate. The mean strain value from this region was used for statistical analysis.

### Histomorphometry.

Histomorphometric analyses were performed using the same femoral or vertebral samples used for micro-CT analysis. Freshly dissected left femurs were fixed in 10% Millonig’s formalin containing 5% sucrose, dehydrated through graded ethanol, and embedded in methyl methacrylate (Sigma–Aldrich). Longitudinal sections (5 μm thick) were prepared for histomorphometric analysis. To quantify osteoclast and osteoblast indices, sections were stained for TRAP (Sigma–Aldrich) and counterstained with toluidine blue (Sigma–Aldrich). The TRAP staining solution consisted of Fast Garnet GBC base, sodium nitrite, naphthol AS-BI phosphate, acetate, and tartrate solutions, and sections were incubated for 105 min at 37°C. Histomorphometric parameters, including osteoclast number (N.Oc/BS), osteoclast surface (Oc.S/BS), osteoclast number per osteoclast perimeter (N.Oc/OcPm), and osteoblast number (N.Ob/BS), were quantified at the femoral endocortical surface using the OsteoMeasure Analysis System (OsteoMetrics, Inc.) coupled to an Olympus BX53 microscope and DP73 digital camera. Analysis was performed in a blinded manner on one section per sample, according to the nomenclature recommended by the American Society for Bone and Mineral Research^21^.

### Serum biomarker analysis.

Whole blood (150–200 μL) was collected from mice by submandibular bleed or cardiac puncture. Serum was isolated by centrifugation at 10,000 × g for 10 min at 4°C. Serum concentrations of the bone resorption marker CTX type I collagen (CTX-I) were measured using a RatLaps CTX-I ELISA kit (Immunodiagnostic Systems, Boldon, UK) and serum concentrations of the bone formation marker P1NP were measured using a Rat/Mouse P1NP EIA kit (Immunodiagnostic Systems) according to the manufacturer’s instructions. Absorbance was read on a multi-mode microplate reader (Cytation 5, BioTek Instruments, Winooski, VT), and sample concentrations were calculated by interpolation from a four-parameter logistic standard curve.

### Bone isocitrate and α-ketoglutarate measurements.

Mouse femurs were harvested, cleaned of adherent soft tissue, and snap-frozen in liquid nitrogen. Bone shafts were pulverized into a fine powder using a pre-chilled mortar and pestle under liquid nitrogen to minimize metabolite degradation. Powdered bone tissue or cell lysates were used to quantify isocitrate and α-ketoglutarate using an Isocitrate Assay Kit (MAK524, MilliporeSigma, St. Louis, MO) and an α-Ketoglutarate Assay Kit (K261, Dojindo Molecular Technologies, Kumamoto, Japan), respectively. Briefly, the assays are based on enzyme-coupled reactions in which isocitrate or α-ketoglutarate is converted into downstream products that generate a measurable signal proportional to metabolite concentration. Assays were performed according to the manufacturers’ protocols, and concentrations were determined by comparison with metabolite standards run in parallel. Signals were measured using a Cytation 5 multi-mode plate reader (BioTek Instruments).

### In vitro osteoclastogenesis.

Murine BMMs were prepared as described previously^13^. Briefly, whole bone marrow cells were flushed from femurs and tibiae of female mice, treated with ACK lysis buffer to remove red blood cells, and plated in α-MEM supplemented with 10% fetal bovine serum, 1% penicillin–streptomycin, and M-CSF (30 ng/mL). After 24 h, non-adherent cells were collected and replated in Petri dishes in the presence of M-CSF (30 ng/mL) for 4 days to generate BMMs, which were used as osteoclast precursors. For osteoclast differentiation, murine BMMs were seeded at 5 × 10^4^ cells per well in 48-well plates and cultured in α-MEM complete medium containing M-CSF (30 ng/mL) and RANKL (30 ng/mL). Mature osteoclasts were generated after 4–5 days, fixed in 10% neutral-buffered formalin for 10 min, and stained for TRAP using a Leukocyte Acid Phosphatase Assay Kit (Sigma–Aldrich) according to the manufacturer’s instructions. Mature osteoclasts were defined as TRAP-positive multinucleated cells containing more than three nuclei. All TRAP staining assays were performed with at least three replicate wells. Representative bright-field and fluorescence images were acquired using an Olympus BX53 microscope equipped with a DP73 digital camera. Osteoclast size was quantified from TdTomato fluorescence images using ImageJ software (NIH). For each condition, images were acquired at ×20 magnification from 10 randomly selected fields per well, and the area of TdTomato-positive multinucleated osteoclasts was measured and averaged to obtain a mean value per well. For metabolite measurements in cultured cells, cell lysates prepared from mature osteoclasts were used to quantify intracellular isocitrate and α-ketoglutarate levels using the same assay kits and procedures described above for bone tissue samples. For pharmacological perturbation experiments, osteoclast cultures were treated with gossypol or enasidenib (Sigma–Aldrich) at the indicated concentrations.

Human BMMs were isolated from bone marrow flushed from femoral heads obtained from female patients undergoing hip replacement surgery. Donors were stratified into young (33, 38, and 46 years old) and aged (70, 76, and 83 years old) groups, and BMMs from donors within each group were pooled for osteoclast differentiation experiments. Donors had no known disorders or medications affecting bone mass or skeletal structure. Bone marrow cells were treated with ACK lysis buffer and plated in α-MEM complete medium containing M-CSF (30 ng/mL) on 100-mm tissue culture dishes. After 24 h, non-adherent cells were collected and further enriched by Ficoll gradient centrifugation to isolate mononuclear hematopoietic cells. These cells were then cultured on 100-mm Petri dishes for 6 days to generate osteoclast progenitors. For osteoclast differentiation, human BMMs were seeded at 2 × 10^5^ cells per well in 48-well plates and cultured with human M-CSF (30 ng/mL) and human RANKL (100 ng/mL). Mature osteoclasts were identified as TRAP-positive multinucleated cells containing more than three nuclei. All human TRAP staining assays were performed in triplicate.

### Caspase-3 activity.

To assess apoptotic susceptibility after osteoclast maturation, osteoclasts derived from young and aged mice were cultured in 6-well plates and harvested 12 h after peak osteoclast formation. For all experiments, this time point was defined relative to the peak of the corresponding control cultures rather than that of each experimental group. Cells were lysed in buffer containing 20 mM Tris-HCl (pH 7.5), 150 mM NaCl, 1 mM EDTA, 10 mM NaF, 1 mM sodium orthovanadate, 1% Triton X-100, and protease inhibitors (5 μg/mL leupeptin, 0.14 U/mL aprotinin, and 1 mM phenylmethylsulfonyl fluoride). Caspase-3 activity was measured by cleavage of the fluorogenic substrate DEVD-AFC (Biomol Research Labs). Briefly, cell lysates were incubated with DEVD-AFC in reaction buffer containing 50 mM HEPES (pH 7.4), 100 mM NaCl, 0.1% CHAPS, 10 mM DTT, 1 mM EDTA, and 10% glycerol. Release of fluorescent AFC was monitored kinetically (excitation/emission, 400/510 nm) using a Cytation 5 microplate reader (BioTek). Protein concentrations were determined using a detergent-compatible DC protein assay (Bio-Rad), and caspase-3 activity was normalized to total protein content.

### Mass spectrometry-based metabolomics.

Untargeted metabolomic profiling was performed on BMMs, pre-osteoclasts (pOCs; 24 h after RANKL stimulation), and mature osteoclasts (mOCs; 3 days after RANKL stimulation), with eight biological replicates per group. Equal numbers of BMMs were seeded for all samples, and the entire cellular material from each well was collected at the indicated time point. Cells were washed twice with ice-cold PBS to remove residual medium, harvested, pelleted, transferred to cryovials, snap-frozen, and stored at − 80°C until shipment on dry ice to Metabolon, Inc. Experimental samples were randomized and balanced across the platform run. Metabolomic profiling was performed using Metabolon’s global metabolomics platform, consisting of a Waters ACQUITY ultrahigh-performance liquid chromatography system coupled to a Thermo Scientific Q Exactive high-resolution Orbitrap mass spectrometer operated at a mass resolution of 35,000. Standard quality-control procedures, compound identification, and peak-area quantification were performed by Metabolon. Median relative standard deviations for internal standards and endogenous metabolites in technical replicates were 6% and 7%, respectively, meeting Metabolon’s quality-control criteria. Metabolite abundances were quantified by area-under-the-curve peak integration. Peak-area data were processed according to Metabolon’s standard procedures, including median scaling within instrument batch, normalization to Bradford protein concentration, minimum-value imputation of missing measurements, and natural-log transformation before downstream analysis.

### Metabolomic data processing and statistical analysis.

Scaled peak intensities were median-scaled within run-day blocks to account for inter-day instrument variation. Metabolites with missing values in ≥ 50% of samples or a standard deviation < 0.25 were excluded before imputation. Remaining missing values were imputed using the minimum observed value for each metabolite, and the resulting abundance values were subsequently log2-transformed to stabilize variance and improve data normality. Differential-abundance analysis was performed using the limma (Linear Models for Microarray Data) framework^22^, which fits a linear model to each metabolite and applies empirical Bayes moderation of variance estimates. Pairwise contrasts were constructed to identify metabolites altered in pOCs and mOCs relative to BMMs and to assess age-associated differences within each osteoclast differentiation stage. Metabolites with a Benjamini–Hochberg-adjusted P value < 0.05 and an absolute log2 fold change > 1.0 were considered significantly altered. Principal component analysis and hierarchical clustering were performed using the processed metabolite-abundance matrix to visualize global differences among experimental groups and relative abundance patterns across samples. For metabolic pathway analysis, significantly increased and decreased metabolites were analyzed separately using MetaboAnalyst 6.0^23^, with KEGG compound identifiers used for metabolite mapping. Pathway results were summarized based on enrichment significance and pathway-impact scores. Metabolon-provided superpathway and subpathway annotations were additionally used to classify altered metabolites and summarize the metabolic processes affected during osteoclast differentiation and aging. Gene Ontology (GO) over-representation analysis of significantly altered metabolites was performed using IDSL.GOA^24^. InChIKey identifiers were used as input to map metabolites through compound–enzyme–gene relationships to metabolic GO terms in the IDSL.GOA knowledgebase. GO-term enrichment was assessed using the platform’s hypergeometric test, with a false-discovery rate cutoff of 0.05.

### Quantitative PCR.

Total RNA was extracted from BMMs, pOCs, and mOCs derived from young, aged, or Atpif1 wild-type and knockout mice, as well as from human osteoclast cultures, using TRIzol reagent (Thermo Fisher Scientific) according to the manufacturer’s instructions. RNA concentration and purity were assessed using a NanoDrop spectrophotometer (Thermo Fisher Scientific). Two micrograms of total RNA were reverse-transcribed using a High-Capacity cDNA Reverse Transcription Kit (Applied Biosystems). Quantitative PCR was performed on a QuantStudio 7 system using TaqMan Universal PCR Master Mix (Thermo Fisher Scientific). The following TaqMan Gene Expression Assays (Applied Biosystems) were used: mouse *Idh1* (Mm00516030_m1), *Idh2* (Mm00612429_m1), *Idh3a* (Mm00499674_m1), *Acp5* (Mm00475698_m1), *Ctsk* (Mm00484039_m1), *Itgb3* (Mm00443980_m1), *Bpgm* (Mm00500291_m1), *Fbxo7* (Mm00462696_m1), *Casp3* (Mm01195085_m1), *Bax* (Mm00432051_m1), *Ripk1* (Mm00436354_m1), *Ripk3* (Mm00444947_m1), *Casp4* (Mm00432304_m1), *Gsdmd* (Mm00509958_m1), *Acsl4* (Mm00490331_m1), *Tfrc* (Mm00441941_m1), and human IDH2 (Hs00953879_m1), IDH3A (Hs00194253_m1), and GAPDH (Hs00266705_g1). Relative mRNA expression was calculated using the ΔCt method after normalization to *Mrps2* (Mm00475528_m1) for mouse samples or GAPDH (Hs00266705_g1) for human samples.

### Mitochondrial respiration and glycolytic ATP production.

Oxygen consumption rate was measured using a Seahorse XF96 Extracellular Flux Analyzer (Agilent Technologies). BMMs, pOCs, and mOCs were analyzed under the indicated experimental conditions. For all assays, 3 × 10^4^ BMMs were seeded per well in XF96 cell culture microplates and cultured under stage-specific differentiation conditions. Before analysis, the growth medium was replaced with Seahorse XF assay medium (Agilent Technologies) supplemented with 10 mM glucose, 1 mM sodium pyruvate, and 2 mM L-glutamine, and cells were equilibrated for 30 min at 37°C in a non-CO_2_ incubator. Mitochondrial respiration was assessed using the Seahorse XF Cell Mito Stress Test. Following baseline measurements, oligomycin (1.5 μM), FCCP (1 μM), and rotenone/antimycin A (0.5 μM each) were sequentially injected to determine ATP-linked respiration, maximal respiratory capacity, and non-mitochondrial respiration, respectively. Basal respiration, ATP-linked respiration, proton leak, and spare respiratory capacity were calculated as previously described^13^. A subset of the Seahorse dataset corresponding to the aged control mature osteoclast group was reported previously^19^ and is included here for comparison with newly generated datasets from additional young and aged cohorts. Real-time ATP production rates derived from mitochondrial respiration and glycolysis were quantified using a Seahorse XF Real-Time ATP Rate Assay Kit (Agilent Technologies) according to the manufacturer’s instructions, following sequential injection of oligomycin and rotenone/antimycin A. Glycolytic ATP production and mitochondrial ATP production were calculated from oxygen consumption rate and proton efflux rate measurements using the manufacturer’s algorithm and normalized to cell number.

### Lentiviral transduction of BMMs.

IDH3A shRNA (mouse) lentiviral particles (sc-62490-V; Santa Cruz Biotechnology) were used for *Idh3a* silencing. Control shRNA Lentiviral Particles-A (sc-108080; Santa Cruz Biotechnology), encoding a non-targeting scrambled shRNA sequence, were used as negative controls. Whole bone marrow cells were isolated from 6- and 24-month-old female C57BL/6 mice as described above. Non-adherent cells were subjected to Ficoll-Hypaque density-gradient centrifugation, and cells recovered from the interface were cultured in the presence of M-CSF (30 ng/ml), polybrene (8 μg/ml; Santa Cruz Biotechnology), and control or *Idh3a* shRNA lentiviral particles for 16 h. Transduced BMMs were then cultured with M-CSF for an additional 24 h, followed by puromycin selection (2 μg/ml; Santa Cruz Biotechnology) for 48 h to remove non-transduced cells. Cells were subsequently used for osteoclast differentiation and downstream analyses as indicated.

### Mitochondrial ROS production.

ROS production was measured using MitoSOX Red reagent (M36008, Thermo Fisher Scientific) according to the manufacturer’s instructions. Osteoclast-lineage cells were seeded in black 96-well plates and cultured under the indicated differentiation conditions. Before measurement, the culture medium was replaced with assay medium containing 3 μM MitoSOX Red, and the cells were incubated for 30 min at 37°C. Fluorescence was measured kinetically for 120 min at excitation/emission wavelengths of 510/580 nm using a Cytation 5 microplate reader (BioTek Instruments), and the rate of MitoSOX oxidation was used as an index of mitochondrial ROS production.

### Western blotting.

BMMs from wild-type and *Atpif1*^ΔLysM^ mice were seeded in 6-well plates and cultured with M-CSF (30 ng/mL) alone or with M-CSF plus RANKL (30 ng/mL) for 3–4 days. Cells were washed twice with ice-cold PBS and lysed in buffer containing 20 mM Tris-HCl (pH 7.4), 150 mM NaCl, and 1% Triton X-100 supplemented with protease and phosphatase inhibitor cocktails (MilliporeSigma, Burlington, MA). Lysates were incubated on ice for 30 min and clarified by centrifugation at 13,000 × g for 15 min at 4°C. Protein concentrations were determined using a DC Protein Assay Kit (Bio-Rad, Hercules, CA). Equal amounts of protein (25 μg per sample) were separated by 10% SDS–PAGE and transferred to PVDF membranes (Bio-Rad). Membranes were blocked in 5% non-fat milk in Tris-buffered saline for 2 h and incubated overnight at 4°C with anti-ATPIF1 antibody (Cell Signaling Technology, #8528) and anti-β-actin antibody (Santa Cruz Biotechnology, sc-81178) as a loading control. After washing, membranes were incubated with HRP-conjugated secondary antibody (Cell Signaling Technology, #7074 or #7076) for 70 min. Signals were detected using enhanced chemiluminescence reagents (MilliporeSigma) and imaged using a VersaDoc system (Bio-Rad).

### Bone resorption activity assay.

BMMs were seeded at 5 × 10^4^ cells per well in calcium phosphate-coated 48-well plates (Cosmo Bio) and cultured in α-MEM complete medium containing M-CSF (30 ng/mL) and RANKL (30 ng/mL) for 4–5 days to induce osteoclast differentiation. Bone resorption activity was assessed by measuring the fluorescence intensity released into the culture medium, according to the manufacturer’s instructions, using FITC-compatible excitation and emission settings on a Cytation 5 microplate reader (BioTek Instruments). After completion of the assay, the remaining medium was carefully removed, and wells were incubated with 5% sodium hypochlorite for 35 min to remove cells. Plates were then washed with water, air-dried, and imaged by light microscopy to visualize resorption pits.

### Statistics.

Statistical analyses were performed using GraphPad Prism 10.0, except for longitudinal BMD data. Normality and homogeneity of variance were assessed before analysis. Longitudinal femoral and vertebral BMD data were analyzed using repeated-measures ANOVA models in SAS version 9.4, including genotype (*Atpif1* wild-type versus conditional knockout), age (months), and their interaction as fixed effects. Based on model fit statistics (AIC and BIC), the final model included categorical effects for genotype, age, and genotype-by-age interaction. For all other experiments, differences between two groups were assessed using unpaired two-tailed Student’s t-tests, whereas comparisons among multiple groups were analyzed by one-way or two-way ANOVA, as appropriate. A *P* value < 0.05 was considered statistically significant.

## Results

### Cortical bone loss with aging is associated with prolonged survival of mature osteoclasts.

We first examined whether osteoclast from young and old mice exhibited different behavior in culture. Bone marrow macrophages (BMMs) from the femurs and tibiae of 4- and 23-month-old mice, cultured with M-CSF and RANKL exhibited similar osteoclast differentiation, which (Fig. 1a) peaked on day 4. As expected, osteoclasts from young mice died rapidly within 24 h after the peak. However, osteoclasts from old mice persisted for at least 72 h (Fig. 1b, c). Young osteoclasts showed increased caspase-3 activity 12 h after peak formation, consistent with enhanced cell death after maturation (Fig. 1d). In contrast, aged osteoclasts exhibited lower caspase-3 activity and reduced expression of genes associated with apoptosis, necroptosis, and ferroptosis, whereas pyroptosis-related markers were unchanged (Fig. 1d and Supplementary Fig. 1a–d). These data suggest that aged osteoclasts acquire broad resistance to regulated cell death programs.

In addition, osteoclasts from aged mice were larger 12 h after peak formation and showed a reduced proportion of mononuclear cells relative to cells from young mice, as assessed using LysM-Cre;TdTomato reporter mice to visualize osteoclast morphology and distinguish mononuclear from multinucleated cells (Fig. 1e–g). Expression of osteoclast markers such as *CathK* and *Acp5* also increased with aging (Fig. 1h). Because mature osteoclasts can undergo fission to generate osteomorphs^25^, we asked whether these changes reflected enhanced osteomorph recycling. However, expression of the osteomorph-associated markers *Bpgm* and *Fbxo7* was not altered in aged osteoclasts (Fig. 1i).

We also differentiated osteoclasts from BMMs obtained from young and aged patients undergoing hip replacement surgery (Supplementary Fig. 2a). Human osteoclast formation peaked on day 6 (Supplementary Fig. 2b–d). Similar to what we observed in mice, osteoclasts from young donors began to decline within 48 h after the peak, whereas those from aged donors persisted for up to 4 additional days and displayed a larger cell size.

We next examined whether osteoclast size was different in 23- versus 4-month-old female mice. As expected, aged mice showed marked endocortical bone loss at both the femoral midshaft and metaphysis, accompanied by increased cortical porosity (Supplementary Fig. 3a–e). Serum markers of bone resorption were elevated with age (Supplementary Fig. 3f). Histomorphometric analysis further showed that osteoclast number and the surface covered by osteoclasts was increased at the endocortical surface in aged mice (Supplementary Fig. 3g–j). Nonetheless, the osteoclast number relative to osteoclast perimeter tended to decrease with aging, indicating that in old mice osteoclast abundance and osteoclast size are increased. Together, these findings suggest that prolonged survival and increased size are conserved features of osteoclasts from aged individuals.

### RANKL induces stage-specific metabolic remodeling during osteoclast differentiation.

To define metabolic changes associated with osteoclast differentiation, we performed untargeted metabolomic profiling of BMMs, pre-osteoclasts (pOCs) collected 24 h after RANKL stimulation, and mature osteoclasts (mOCs) collected at day 3 cultured from 4-month-old female C57BL/6 mice (Fig. 2a, b). Principal component analysis revealed clear separation among BMMs, pOCs, and mOCs, indicating distinct metabolic states across differentiation (Fig. 2c). Differential metabolite analysis identified 399 metabolites altered across these stages, including 31 shared changes, 25 unique to early differentiation, 6 specific to late differentiation, and 164 associated with maturation from progenitors to multinucleated osteoclasts (Fig. 2d). These data indicate that RANKL drives broad and stage-resolved metabolic remodeling during osteoclastogenesis.

To examine early metabolic remodeling, we compared BMMs and pOCs from 4-month-old female mice. Volcano-plot analysis showed that AMP, reduced glutathione (GSH), itaconate, and NAD^+^ were increased in pOCs, whereas L-pyrroline-5-carboxylate, cystine, and α-ketoglutarate were decreased (Fig. 2e). Pathway-enrichment analysis highlighted methionine and glutamate metabolism together with short-, medium-, and long-chain β-oxidation-related pathways (Fig. 2f), consistent with coordinated remodeling of redox-associated and mitochondrial metabolism during early osteoclast commitment. Hierarchical clustering confirmed elevation of AMP, GSH, NAD^+^, and cysteinylglycine in pOCs, whereas several glycolytic and amino-acid-associated intermediates were reduced (Supplementary Fig. 4a). Pathway-network analysis further showed that upregulated metabolites clustered predominantly within oxidative and redox-related pathways, with nucleotide-associated pathways forming a partially connected but distinct module (Supplementary Fig. 4b). Together, these data support an early metabolic transition characterized by redox remodeling and increased mitochondrial pathway engagement after RANKL stimulation.

We next examined metabolic remodeling during osteoclast maturation by comparing mOCs with BMMs from 4-month-old female mice. Mature osteoclasts showed increased AMP, xanthosine monophosphate, GSH, coenzyme A, NAD^+^, and nicotinamide riboside, whereas dihydroxyacetone phosphate, 2—*UMP*, 2-deoxyinosine, and 3`-GMP were decreased (Fig. 2g). Pathway-enrichment analysis highlighted NAD metabolism, CoA biosynthesis, and other cofactor-linked mitochondrial pathways (Fig. 2h), consistent with coordinated remodeling of redox and acyl-carrier metabolism during osteoclast maturation. This pattern was supported by heatmap analysis, which confirmed elevation of AMP, xanthosine monophosphate, GSH, CoA, NAD^+^, and nicotinamide riboside in mOCs, together with reduction of glycolytic and nucleotide catabolic intermediates (Supplementary Fig. 5a). Network analysis similarly identified clustered enrichment of NAD^+^- and CoA-associated pathways, indicating integration of bioenergetic, antioxidant, and biosynthetic programs in mature osteoclasts (Supplementary Fig. 5b).

To further assess metabolites decreased during differentiation, we performed pathway-enrichment analysis of downregulated metabolite sets in both pOCs and mOCs. Although the statistical significance was modest, glycolysis-related pathways, including the Warburg effect and gluconeogenesis-associated signatures, were consistently reduced in both comparisons (Supplementary Fig. 4c, 5c). Together, these findings define a stage-resolved metabolic program during osteoclastogenesis, characterized by coordinated reduction of glycolysis-associated metabolites and enrichment of mitochondrial and cofactor-linked pathways, and provide a framework for examining how aging reshapes osteoclast metabolism.

### Aging reprograms mature osteoclast metabolism toward a glycolysis-associated state.

We next compared BMMs, pOCs and mOCs generated from 4- and 23-month-old female C57BL/6 mice. Principal component analysis revealed age-dependent separation in BMMs and mOCs, whereas pOCs showed comparatively limited divergence, indicating that aging-associated metabolic remodeling is most evident in progenitors and mature osteoclasts (Fig. 3a). Consistent with this, Venn analysis identified age-regulated metabolites that were largely specific to BMMs and mOCs (Fig. 3b). In aged mOCs, citrate, isocitrate, aconitate, 2-phosphoglycerate, and phosphoenolpyruvate were increased relative to young controls (Fig. 3c, d). Because these metabolites span glycolytic and TCA-linked nodes, these data indicate remodeling at the interface between glycolysis and mitochondrial metabolism in aged osteoclasts.

Pathway-enrichment and network analyses further identified Warburg effect, glycolysis, and gluconeogenesis-associated pathways among the most enriched metabolic programs in aged osteoclasts (Fig. 3e, f). Because these inferences are based on metabolite abundance rather than direct tracer-based flux measurements, they do not establish pathway flux per se. Nevertheless, the coordinated increase in glycolysis-associated intermediates supports a shift in metabolic state toward greater glycolytic engagement in aged mOCs. Consistent with this interpretation, enrichment analysis of metabolites increased in aged mOCs identified oxaloacetate metabolism (Supplementary Fig. 6a), whereas metabolites decreased in aged mOCs were enriched for pathways associated with fatty-acid oxidation, glutamate metabolism, and pyruvate metabolism (Supplementary Fig. 6b). Together, these data indicate an age-associated metabolic remodeling in mature osteoclasts, characterized by increased glycolysis-associated signatures with relative reduction of several mitochondrial fuel-utilization pathways.

To validate these metabolomic changes, we measured intracellular metabolites and found that isocitrate was increased in both cultured osteoclasts and bone tissue from aged mice (Fig. 3g). α-ketoglutarate, the downstream product of isocitrate oxidation (Supplementary Fig. 8a), was increased in aged osteoclasts, with a similar but non-significant trend observed in bone tissue (Fig. 3h). Finally, glycolysis-linked respiration was markedly increased in osteoclasts from aged mice, and this age-associated increase was strongly attenuated by conditional deletion of the mitochondrial deacetylase *Sirt3* (Fig. 3i). In contrast, *Sirt3* deletion had no detectable effect in osteoclasts from young mice. These findings indicate that aging drives glycolysis-coupled metabolic remodeling at the interface with the TCA cycle in mature osteoclasts. Given the coordinated changes in isocitrate and α-ketoglutarate, we next examined the enzymatic machinery governing this metabolic node.

### IDH3A is selectively induced in aged mature osteoclasts and supports glycolysis-coupled metabolic remodeling.

Because age-associated metabolite changes converged on the isocitrate– α-ketoglutarate node, we next examined IDH enzymes that could sustain this program (Supplementary Fig. 8a). Among the three IDH subunits, *Idh3A*, which encodes the catalytic subunit required for IDH3A activity, was selectively increased in mature osteoclasts from aged, but not young, female C57BL/6 mice during osteoclastogenesis (Fig. 4a). By contrast, the NADP-dependent isoforms *Idh1* and *Idh2* were not induced by RANKL in aged cells (Supplementary Fig. 8b). Consistent with this, hIDH3 was also increased in osteoclasts from aged human donors (Supplementary Fig. 2e).

We next examined the bioenergetic consequences of this age-associated increase in IDH3A. Glycolytic ATP production was markedly increased in mature osteoclasts from aged mice, whereas the effect in young cells was minimal (Fig. 4b). Mitochondrial ATP production also increased during osteoclastogenesis, but to a lesser extent in aged osteoclasts, consistent with a relative shift toward glycolytic ATP dependence. To provide genetic support for a functional role of IDH3A, we suppressed *Idh3a* expression using shRNA during osteoclast differentiation. *Idh3a* knockdown reduced glycolytic ATP production and osteoclast formation in aged, but not young, osteoclast cultures (Fig. 4c, d). Consistent with a loss of the enhanced survival phenotype observed in aged osteoclasts, *Idh3a* knockdown also increased caspase-3 activity in aged cells (Fig. 4e). These findings support a role for IDH3A-associated metabolic remodeling in sustaining glycolytic ATP production, osteoclast formation, and the enhanced survival phenotype associated with aging. Because genetic suppression during differentiation cannot distinguish effects on osteoclast formation from those on mature osteoclast function, we next used a pharmacological approach in fully differentiated osteoclasts. Gossypol, which has been reported to perturb IDH3-associated metabolism^26,27^, was administered after peak osteoclast formation to determine whether disruption of this metabolic state also affects established osteoclasts (Fig. 4f). Gossypol markedly decreased osteoclast number, with a stronger effect in aged cells, prevented the age-associated increase in resorption (Fig. 4g), and abolished glycolysis-derived ATP production in aged, but not young, osteoclasts (Fig. 4h). Gossypol also increased caspase-3 activity in aged mature osteoclasts, consistent with reduced survival (Fig. 4i).

To determine whether the age-dependent response was specific to perturbation of IDH3A-associated metabolism rather than a general consequence of inhibiting mitochondrial IDH activity, we compared the effects of gossypol with those of the IDH2 inhibitor enasidenib. Although enasidenib inhibited osteoclast formation in a dose-dependent manner, its effects were comparable between young and aged cells (Supplementary Fig. 8c), in contrast to the greater inhibitory effect of gossypol in aged osteoclasts. Notably, gossypol did not alter mitochondrial reactive oxygen species (ROS) levels in either BMMs or mature osteoclasts (Fig. 4j), indicating that its effect in aged osteoclasts was not associated with altered mitochondrial ROS accumulation. Together, these findings identify IDH3A as a selective metabolic regulator in aged mature osteoclasts and support a model in which IDH3A-dependent coupling of glycolysis-associated metabolism to the TCA cycle sustains osteoclast survival and function during aging.

### ATPIF1 regulates the IDH3A-associated glycolysis-coupled metabolic program in osteoclasts during skeletal aging.

Because ATPIF1 has been implicated in glycolytic adaptation and cell survival under conditions of high energetic demand^28,29^ and was identified in our previous acetylome analysis as a mitochondrial SIRT3 target^19^, we next examined its role in osteoclast metabolism during skeletal aging. We generated osteoclast-lineage-specific *Atpif1* conditional knockout mice (*Atpif1^ΔLysM^* mice) and first confirmed that ATPIF1 protein was induced by RANKL in control BMMs and efficiently deleted in *Atpif1^ΔLysM^* BMMs (Fig. 5a).

We then aged female *Atpif1^ΔLysM^* mice and littermate controls to 18 months and longitudinally monitored skeletal phenotypes. Serial dual-energy X-ray absorptiometry (DXA) analysis showed that control mice reached peak bone mass at approximately 6 months of age and start losing bone with aging after 12 months of age (Fig. 5b, c). Bone mineral density (BMD) in *Atpif1^ΔLysM^* mice was similar to that of controls up to 12 months of age. However, deletion of *Atpif1* attenuated age-related loss of femoral BMD but not spinal BMD. Consistent with this, micro-computed tomography (micro-CT) analysis showed that cortical architecture, cortical porosity, and spinal trabecular parameters were comparable between genotypes at 6 months of age (Fig. 5d–h). With aging, control mice exhibited marked cortical thinning at both the femoral midshaft and metaphysis, accompanied by expansion of total and medullary areas (Fig. 5d, e and Supplementary Fig. 9a–d). These age-associated cortical changes were substantially attenuated in *Atpif1^ΔLysM^* mice, including reduced medullary area expansion (Supplementary Fig. 9b, d) and reduced cortical porosity (Fig. 5f). By contrast, trabecular bone loss in the femur and spine was similar between genotypes (Fig. 5g, h and Supplementary Fig. 9e–j). Digital volume correlation analysis further showed a trend toward greater bone strength in aged *Atpif1^ΔLysM^* mice, as indicated by reduced von Mises strain under identical loading conditions (Fig. 5i). Consistent with preservation of cortical structure, aged *Atpif1^ΔLysM^* mice had lower serum C-terminal telopeptide (CTX), a marker of bone resorption, whereas procollagen type I N-terminal propeptide (P1NP), a marker of bone formation, was unchanged (Fig. 5j). To define the cellular basis of these phenotypes, we performed histomorphometric analysis of the endocortical surface. Osteoclast number was comparable between genotypes (Fig. 6a). However, *Atpif1* deletion reduced the surface of bone covered by osteoclast and increased the osteoclast number relative to osteoclast perimeter, suggesting reduced osteoclast size on the endocortical surface.

We next asked whether ATPIF1 contributes to the glycolysis-coupled metabolic program identified in aged osteoclasts. Cultured osteoclasts from BMMs of aged mice revealed that deletion of *Atpif1* reduced expression of several IDH family members, with the most prominent suppression observed for *Idh3a* (Fig. 6b). Consistent with this, α-ketoglutarate levels were reduced in osteoclasts from aged *Atpif1^ΔLysM^* mice (Fig. 6c). This was accompanied by impaired osteoclast differentiation, reduced expression of functional osteoclast markers including *Itgb3* and *Ctsk*, and lower resorptive activity on calcium phosphate-coated plates (Fig. 6d–f). These effects were not observed in young *Atpif1^ΔLysM^* osteoclasts (Supplementary Fig. 10a–c). Deletion of *Atpif1* reduced glycolysis-derived ATP production and mitochondrial ROS in aged osteoclasts (Fig. 6g, h), whereas modest increases in these parameters were observed in young osteoclasts (Supplementary Fig. 10d). In parallel with the reduction in osteoclast formation, caspase-3 activity was increased in aged *Atpif1^ΔLysM^* osteoclasts (Fig. 6i). Together, these findings identify ATPIF1 as an upstream regulator of the IDH3A-associated glycolysis–TCA metabolic program that supports osteoclast formation, survival, and resorptive function during skeletal aging.

### Pharmacological targeting of IDH3A-linked metabolism attenuates age-related cortical bone loss.

We next asked whether pharmacological disruption of Idh3a could alter age-related bone loss. Gossypol was administered to 18-month-old female C57BL/6 mice for 12 weeks (Fig. 7a). Gossypol reduced α-ketoglutarate levels in bone tissue (Fig. 7b) and improved cortical bone parameters, including an increase in cortical thickness at both the femoral midshaft and metaphysis and a reduction in cortical porosity (Fig. 7c). Consistent with these structural changes, serum CTX levels were reduced in gossypol-treated mice, whereas P1NP levels trended upward (Fig. 7d, e). Histomorphometric analysis of the femoral endocortical surface further showed a reduction in osteoclast number per bone perimeter, whereas osteoblast number, osteoclast surface per bone surface, and osteoclast number relative to osteoclast perimeter were not significantly altered (Supplementary Fig. 11). Together, these findings indicate that pharmacological targeting of IDH3A-linked metabolism attenuates age-related cortical bone loss *in vivo* and support the therapeutic potential of this metabolic pathway in skeletal aging.

## Discussion

Our findings indicate that skeletal aging is associated with marked metabolic remodeling in osteoclasts, characterized by increased glycolytic ATP production together with coordinated changes in metabolites linked to the early TCA cycle. This pattern is consistent with prior evidence that osteoclast differentiation and function are accompanied by increased glycolytic engagement, and that mature osteoclasts can rely heavily on aerobic glycolysis despite the presence of oxygen^12,30^. In our model, however, this state is more accurately described as a glycolysis-coupled TCA cycle program rather than a simple shift away from mitochondrial metabolism. Indeed, the accumulation of isocitrate and α-ketoglutarate in aged osteoclasts suggests remodeling at a metabolic node that links glycolysis to the TCA cycle and may help sustain the energetic and functional demands of mature osteoclasts. Notably, although only a limited number of metabolites were significantly altered between young and aged osteoclasts, these changes were functionally convergent and centered on glycolysis-linked entry into the TCA cycle. This pattern suggests that aging does not broadly rewire osteoclast metabolism, but instead induces a focused energetic reprogramming that favors glycolytic dependence while preserving selected mitochondrial-linked functions. Thus, our findings extend earlier work on osteoclast bioenergetics by showing that aging causes not only altered osteoclast respiration, but also by a distinct reorganization of carbon metabolism that supports prolonged survival and sustained resorptive function.

Although osteoclasts from aged mice displayed a clear glycolytic bias, several mitochondrial and redox-associated metabolites, including NAD^+^ and glutathione, remained elevated relative to macrophages (Supplementary Fig. 5). This suggests that mitochondrial and redox metabolism may still be engaged alongside glycolysis rather than being uniformly suppressed. This pattern may reflect a compensatory adaptation to the high energetic and oxidative demands of mature osteoclasts, in which glycolytic ATP production is accompanied by selective preservation of metabolite pools that support mitochondrial function and redox balance. In this context, citrate and itaconate may be of particular interest. Citrate is increasingly recognized as a central metabolic node that can couple carbon metabolism to lipid biosynthesis and membrane remodeling. In contrast, itaconate has emerged as a regulator of stress-responsive metabolism through inhibition of succinate dehydrogenase and rewiring of TCA-linked flux^31–33^.

Although the specific functions of these metabolites in osteoclasts from aged mice remain to be determined, their enrichment is consistent with the idea that metabolic remodeling in these cells is not simply a unidirectional shift away from mitochondrial metabolism, but rather a hybrid state in which glycolytic and mitochondrial-redox programs coexist to sustain osteoclast survival and function under aging-related stress. Indeed, analogous hybrid metabolic states have been described in other cell systems, including cancer cells, in which enhanced aerobic glycolysis coexists with preserved mitochondrial and redox metabolism to sustain survival and stress adaptation^16,34,35^.

Although the present study focused primarily on metabolic remodeling in mature osteoclasts, our metabolomic profiling also suggests that aging-associated changes affect osteoclast precursor cells. In BMMs from aged mice, a larger number of metabolites were altered than in mature osteoclasts, with enrichment of pathways related to glutamate, glutathione, purine, pyrimidine, and other amino acid metabolism (Supplementary Fig. 7). Network analysis further highlighted a glutamate–aspartate–cysteine–glutathione–methionine axis, pointing to coordinated remodeling of amino acid, redox, and mitochondrial-linked metabolism. These features are consistent with the possibility that aged osteoclast progenitors are not metabolically neutral precursors, but instead exist in a preconditioned state that may favor survival, stress adaptation, and eventual osteoclastogenic commitment. These findings raise the possibility that aging reprograms osteoclast metabolism at multiple stages of the lineage, with intrinsic metabolic priming in BMMs setting the stage for the glycolysis-coupled program that later emerges in mature osteoclasts. Defining how this early progenitor remodeling contributes to age-related osteoclastogenesis will be an important goal for future studies.

Deletion of *Atpif1* attenuated age-related femoral bone loss, preserved cortical structure, reduced cortical porosity, and lowered serum CTX without altering P1NP, supporting a direct effect on bone resorption. At the endocortical surface, osteoclast number was unchanged, whereas osteoclast surface per bone surface was reduced and osteoclast number relative to osteoclast perimeter was increased, consistent with smaller osteoclast size and diminished resorptive output per osteoclast. Together with the *ex vivo* findings that *Atpif1* deletion reduced *Idh3a* expression, α-ketoglutarate accumulation, glycolytic ATP production, and resorptive activity in aged osteoclasts, these results place ATPIF1 as a key mediator linking mitochondrial stress signaling to osteoclast function during skeletal aging.

In contrast to the near-complete protection against age-related endocortical bone loss achieved by genetic or pharmacologic inhibition of *Sirt3*^13,19^, the skeletal phenotype produced by *Atpif1* deletion was milder, likely reflecting both biological and technical factors. Biologically, SIRT3 acts as a broad mitochondrial deacetylase^36^, and our previous studies identified multiple SIRT3-sensitive mitochondrial targets in osteoclasts in addition to ATPIF1^19^, indicating that ATPIF1 is an important downstream effector but unlikely to account for the full skeletal output of the SIRT3 pathway. This interpretation is consistent with the stronger protection produced by *Sirt3* loss in aging models, particularly in female mice and more prominently in cortical than trabecular bone. Several non-mutually exclusive explanations may underlie this sex and compartment specificity. First, age-related cortical bone loss in mice is driven to a large extent by endocortical and intracortical remodeling, processes that are especially dependent on osteoclast activity and osteocyte-derived RANKL, whereas trabecular bone loss with aging is influenced by additional mechanisms, including impaired bone formation^6,37,38^. Second, our previous studies showed that perturbation of osteoclast mitochondrial function through SIRT3 has more pronounced skeletal consequences in aged female than male mice^13,19^, suggesting that mitochondrial control of osteoclast activity may be particularly important in the female aging skeleton. Thus, the SIRT3–ATPIF1 axis may become most consequential under conditions in which osteoclast mitochondrial adaptation contributes strongly to ongoing bone resorption, such as aged female cortical bone. A limitation of the present study is that LysM-Cre targets the broader myeloid lineage rather than mature osteoclasts selectively^39,40^. Therefore, the relative contributions of ATPIF1 during osteoclast differentiation versus in fully differentiated osteoclasts cannot be resolved with this model. Future studies using more selective mature-osteoclast Cre drivers will help define the stage-specific role of ATPIF1 and distinguish ATPIF1-mediated effects from other SIRT3-dependent pathways.

Several additional limitations of the present study should be noted. First, although our data place ATPIF1 upstream of an IDH3A-associated glycolysis-coupled metabolic program, the mechanistic relationship between these two proteins remains incompletely defined. In the current study, *Atpif1* deletion reduced *Idh3a* expression in aged osteoclasts, but we did not directly determine whether ATPIF1 regulates IDH3A activity, stability, or metabolic flux. Thus, ATPIF1 should be viewed here as a regulator of an IDH3A-associated program rather than as a definitively established molecular activator of IDH3A itself. This distinction is important because our earlier acetylomic analyses showed that SIRT3 remodels multiple mitochondrial targets in osteoclasts^19^, indicating that ATPIF1 is likely a major, but not exclusive, downstream effector of the broader SIRT3 pathway. Second, our findings suggest that ATPIF1 influences a broader mitochondrial stress-response network than the IDH3A-associated metabolic arm alone. Genetic deletion of *Atpif1* reduced mitochondrial ROS in aged osteoclasts, whereas pharmacologic perturbation with gossypol suppressed glycolytic ATP production without altering mitochondrial ROS. This distinction is consistent with previous work showing that ATPIF1 can promote glycolytic reprogramming through altered ATP synthase activity, mitochondrial ROS accumulation, and HIF1α stabilization^29^. It is also consistent with recent evidence in bone marrow erythroblasts showing that *Atpif1* deficiency impairs cell proliferation together with mitochondrial OXPHOS and TCA-cycle function^41^. Together with the effects of shRNA-mediated *Idh3a* suppression, these findings support the involvement of IDH3A in the glycolytic component of this metabolic program while suggesting that ATPIF1 exerts broader effects on mitochondrial bioenergetic and redox state. The precise causal relationships among ATPIF1, mitochondrial ROS, and IDH3A in aged osteoclasts remain to be established. Finally, direct *in vivo* validation of glycolytic remodeling remains incomplete. Although we detected increased isocitrate and α-ketoglutarate in bone shaft samples from aged mice, these measurements were obtained from osteoclast-enriched bone tissue rather than from purified osteoclasts in situ. Because bone shaft preparations contain multiple cell populations in addition to osteoclasts, these findings support *in vivo* relevance but do not establish osteoclast-specific metabolic remodeling with certainty. Future studies using cell-resolved *in vivo* metabolite measurements, metabolic tracing, and more selective mature osteoclast targeting strategies will be needed to define the full architecture of the SIRT3–ATPIF1–IDH3A axis in skeletal aging.

Together, these findings support a model in which aging drives a selective metabolic reprogramming in osteoclasts, characterized by a glycolysis-coupled TCA program that promotes bone resorption. Rather than reflecting a simple shift away from mitochondrial metabolism, this adaptive state appears to involve coordinated engagement of glycolytic, mitochondrial, and redox-linked pathways in response to aging-associated stress. These results expand the current framework for osteoclast bioenergetics and identify the SIRT3–ATPIF1–IDH3A axis as a candidate pathway linking mitochondrial stress adaptation to cortical bone loss with aging. Future studies will be needed to determine whether targeting this metabolic program can be translated into therapeutic strategies for age-associated skeletal fragility.

## Supplementary Material

This is a list of supplementary files associated with this preprint. Click to download.

- SupplementalFigure9.tif
- SupplementalFigure5continued.tif
- SupplementalFigure8.tif
- SupplementalFigure2.tif
- SupplementalFigure7continued1.tif
- SupplementalFigure7continued2.tif
- SupplementalFigure6.tif
- SupplementalFigure3.tif
- SupplementalFigure4.tif
- SupplementalFigure10.tif
- SupplementalFigure5.tif
- SupplementalFigure1.tif
- SupplementalFigure11.tif
- SupplementalFigure7.tif
- SupplementaryInformation.pdf

## Figures and Tables

**Figure 1:**
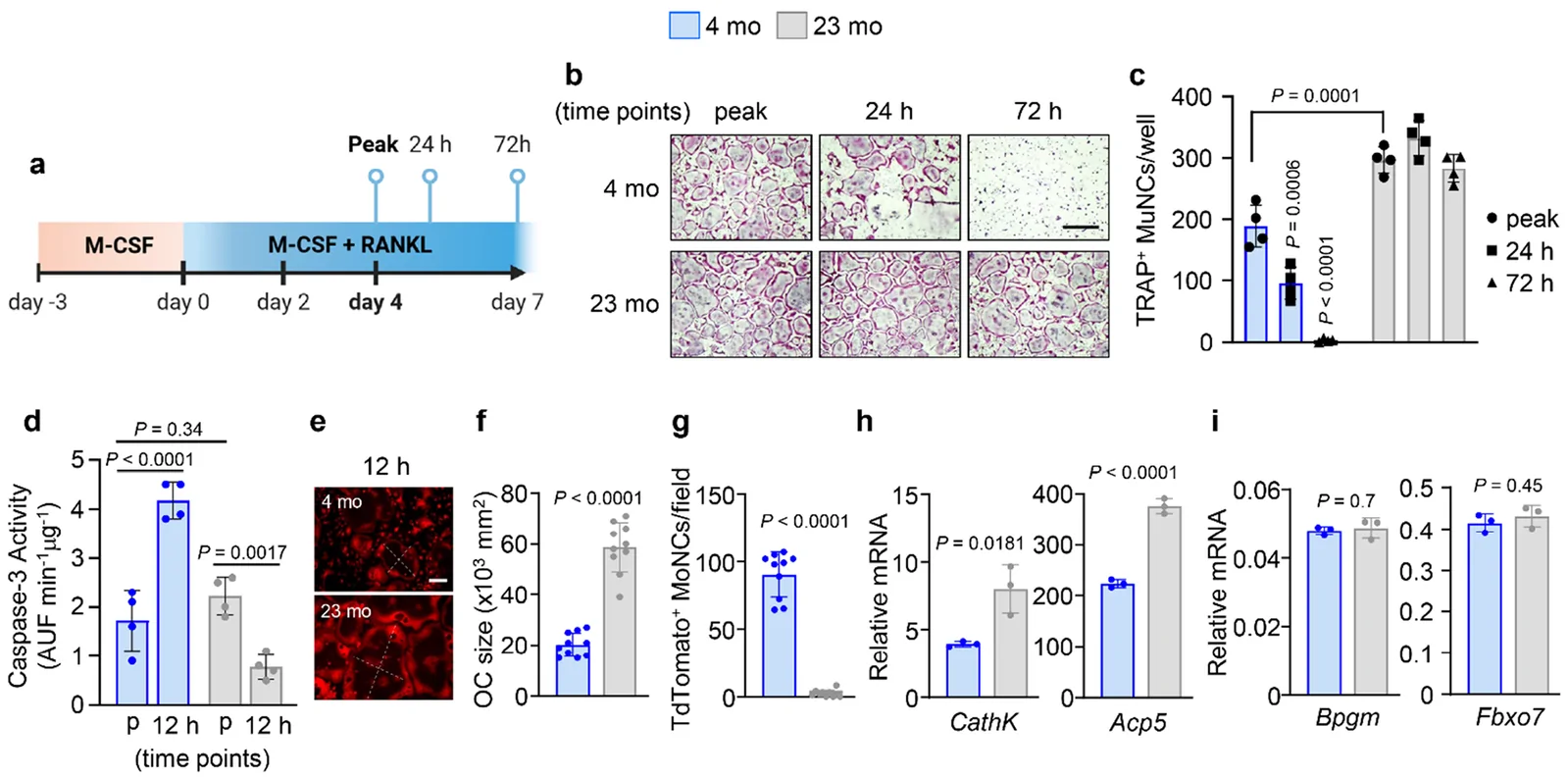
Aged osteoclasts exhibit prolonged survival and sustained resorptive features after peak formation. (**a**) Schematic of the *in vitro* osteoclast differentiation and post-maturation analysis workflow. BMMs isolated from femurs and tibiae of 4- and 23-month-old C57BL/6 female mice were cultured with M-CSF and RANKL, and analyses were performed at peak osteoclast formation and at the indicated time points thereafter. (**b**) Representative TRAP staining images of osteoclast cultures generated from 4- and 23-month-old mice at peak formation, and 24 h and 72 h after the peak time point. (**d**) Quantification of TRAP-positive multinucleated cells (MuNCs) per well at the indicated time points. (**d**) Caspase-3 activity in osteoclast cultures at peak formation and 12 h later. (**e**) Representative TdTomato fluorescence images of osteoclasts from 4- and 23-month-old mice at 12 h after peak formation. (**f**) Quantification of osteoclast size from TdTomato fluorescence images. (**g**) Quantification of TdTomato-positive mononuclear cells. (**h, i**) Relative mRNA expression of the indicated mature osteoclast or osteomorph marker genes in osteoclasts generated from 4- and 23-month-old mice. Data are means ± s.d. Statistical analyses were performed using unpaired two-tailed Student’s t-test or one-way/two-way ANOVA, as appropriate. Exact *P* values are shown in the panels. Representative images are from at least three independent experiments. Scale bars, 500 mm. All measures were performed in cultured BMMs pooled from 4–5 mice/group.

**Figure 2:**
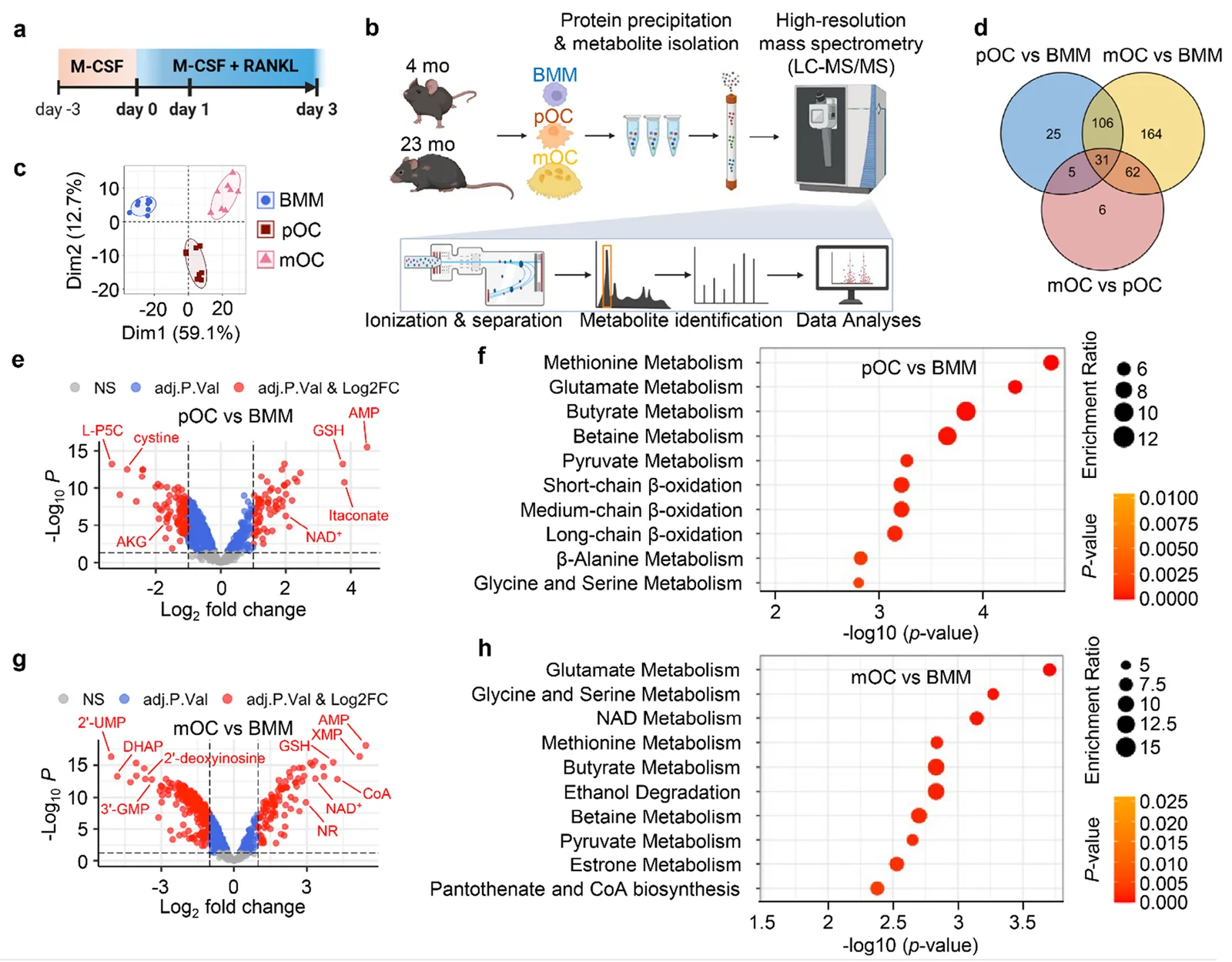
Metabolomic profiling during osteoclast differentiation. (**a**) Experimental scheme for metabolomic profiling during osteoclast differentiation. BMMs were generated with M-CSF, followed by RANKL stimulation to generate 24-h pre-osteoclasts (pOCs) and day 3 mature osteoclasts (mOCs). (**b**) Schematic of sample collection from 4- and 23-month-old female mice and the workflow for metabolite extraction and LC–MS/MS-based untargeted metabolomics analysis. (**c-h**) Metabolomic analyses of BMMs, pOCs, and mOCs derived from 4-month-old female mice in response to RANKL-induced osteoclast differentiation. (**c**) Principal component analysis (PCA) of metabolomic profiles. (**d**) Venn diagram showing the overlap of significantly altered metabolites among BMMs, pOCs, and mOCs. (**e, g**) Volcano plots of differentially abundant metabolites in pOCs (**e**) and mOCs (**g**) relative to BMMs. Selected metabolites are highlighted. (**f, h**) Pathway-enrichment analysis of metabolites altered in pOCs (**f**) and mOCs (**h**) relative to BMMs. Dot size indicates enrichment ratio, and colour indicates *P*value. Criteria for differential metabolite abundance and pathway-enrichment analysis are described in the Methods. All measures were performed in cultured BMMs pooled from 4–5 mice/group.

**Figure 3:**
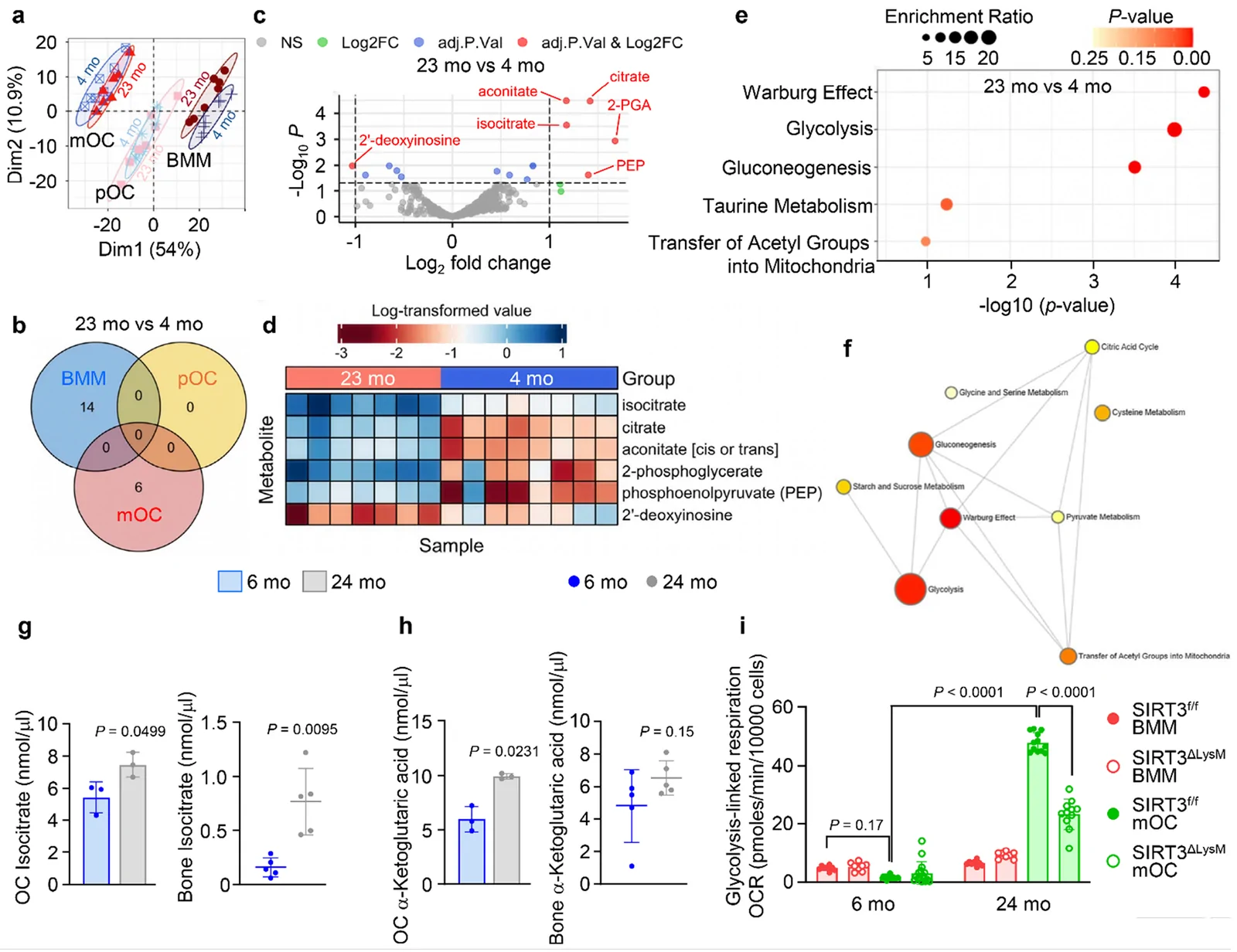
Metabolomic profiling of mature osteoclasts during skeletal aging. (**a, b**) Metabolomic analyses of bone marrow macrophages (BMMs), pre-osteoclasts (pOCs), and mature osteoclasts (mOCs) derived from 4- and 23-month-old female mice. (**a**) Principal component analysis of metabolomic profiles. (**b**) Venn diagram showing the overlap of significantly altered metabolites. (**c–f**) Metabolomic analyses of mOCs from 4- and 23-month-old female mice. (**c**) Volcano plot of differentially abundant metabolites. Selected metabolites are highlighted. (**d**) Heat map of representative metabolites altered in mOCs. (**e, f**) Pathway-enrichment (**e**) and network (**f**) analyses of metabolites altered in mOCs. Dot size indicates enrichment ratio, and colour indicates *P* value in (**e**). (**g, h**) Validation of selected TCA-linked metabolites in osteoclasts and bone shaft samples from 6- and 24-month-old female mice. (**g**) Isocitrate levels in cultured osteoclasts (*left*) and bone shaft samples (*right*). (**h**) a-ketoglutarate levels in cultured osteoclasts (*left*) and bone shaft samples (*right*). (**i**) Glycolysis-linked respiration in BMMs and mOCs from 6- and 24-month-old control and osteoclast-lineage *Sirt3*-deficient mice. A subset of the Seahorse data shown in this panel, corresponding to the aged control mature osteoclast group, was previously reported in Richardson et al., Mol Metab, 2024^19^. These data are included here for comparison and were reanalyzed together with newly generated datasets from additional young and aged groups in the context of aging-associated metabolic remodeling. Criteria for differential metabolite abundance and pathway-enrichment analysis are described in the Methods. Data are means ± s.d. Statistical analyses were performed using unpaired two-tailed Student’s t-test or two-way ANOVA, as appropriate. Exact *P* values are shown in the panels. All measures were performed in cultured BMMs pooled from 4–5 mice/group.

**Figure 4:**
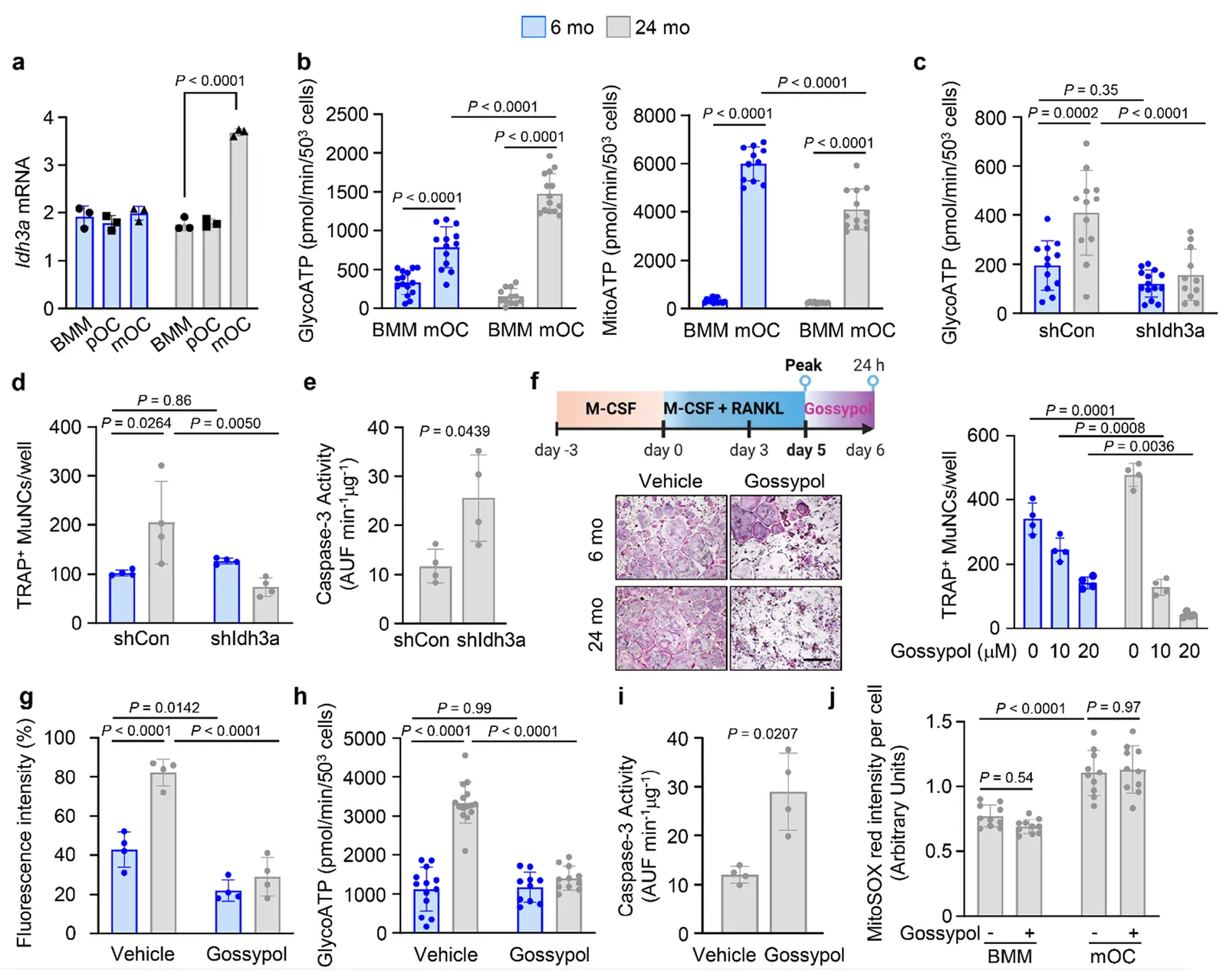
IDH3A-linked metabolism supports osteoclast function during aging. (**a, b**) Analyses of Idh3a expression and ATP production in BMMs and mOCs from 6- and 24-month-old female mice. (**a**) Relative *Idh3a* mRNA expression. (**b**) Glycolytic ATP production (*left*) and mitochondrial ATP production (*right*). (**c–e**) Effects of shRNA-mediated *Idh3a*suppression in osteoclast cultures generated from BMMs of 6- and 24-month-old female mice. (**c**) Glycolytic ATP production in mOCs transduced with shCon or shIdh3a. (**d**) Quantification of TRAP-positive multinucleated cells (MuNCs) per well in osteoclast cultures transduced with shCon or shIdh3a. (**e**) Caspase-3 activity in aged osteoclast cultures transduced with shCon or shIdh3a. (**f–j**) Effects of gossypol on osteoclast function and metabolism. (**f**) Experimental scheme for post-maturation gossypol treatment (*up left*). Representative TRAP staining images (*down left*) and quantification of TRAP-positive MuNCs per well (*right*) in osteoclast cultures treated with vehicle or gossypol. (**g**) Quantification of resorption activity in osteoclast cultures treated with vehicle or gossypol (20 mM). (**h**) Glycolytic ATP production in mOCs treated with vehicle or gossypol (20 mM). (**i**) Caspase-3 activity in aged osteoclast cultures treated with vehicle or gossypol (20 mM). (**j**) MitoSOX Red fluorescence intensity per cell in BMMs and mOCs treated with vehicle or gossypol (20 mM). Data are means ± s.d. Statistical analyses were performed using unpaired two-tailed Student’s t-test or two-way ANOVA, as appropriate. Exact *P*values are shown in the panels. Representative images are from at least three independent experiments. Scale bars, 500 mm. All measures were performed in cultured BMMs pooled from 4–5 mice/group.

**Figure 5:**
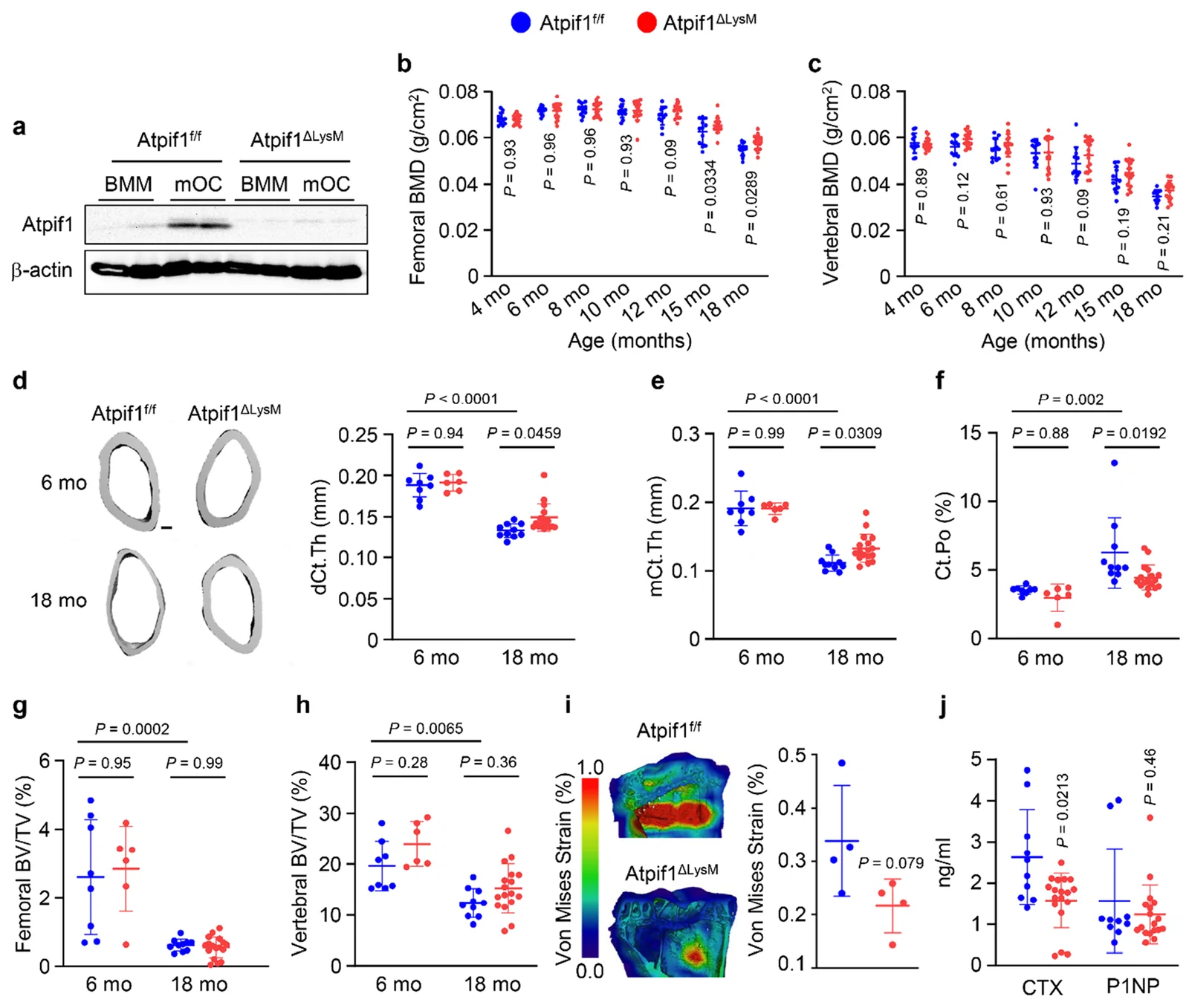
Deletion of *Atpif1* in the osteoclast lineage attenuates age-related cortical bone loss. (**a**) Immunoblot of ATPIF1 protein in BMMs and mOCs from control and *Atpif1*^ΔLysM^ mice. Each lane represents cells derived from an individual mouse. b-actin was used as a loading control. (**b, c**) Longitudinal DXA analysis of femoral (**b**) and vertebral (**c**) BMD in control and *Atpif1*^ΔLysM^ mice. (**d–h**) Micro-CT–based analysis of cortical and trabecular bone parameters in control and *Atpif1*^ΔLysM^ mice at 6 and 18 months of age. (**d**) Representative cross-sectional micro-CT images and cortical thickness at the femoral diaphysis (dCt.Th). Scale bars, 100 mm. (**e**) Cortical thickness at the femoral metaphysis (mCt.Th). (**f**) Cortical porosity (Ct.Po). (**g**) Femoral trabecular bone volume fraction (BV/TV). (**h**) Vertebral trabecular bone volume fraction (BV/TV). (**i**) Digital volume correlation analysis of tibial von Mises strain under compressive loading in control and *Atpif1*^ΔLysM^ mice. Representative strain maps are shown on the left, with quantification on the right. (**j**) Serum CTX and P1NP levels in 18-month-old control and *Atpif1*^ΔLysM^ mice. Data are means ± s.d. Statistical analyses were performed using unpaired two-tailed Student’s t-test, repeated-measures ANOVA, or two-way ANOVA, as appropriate. Exact *P* values are shown in the panels. For *in vivo* skeletal phenotyping, n = 6–17 animals/group.

**Figure 6:**
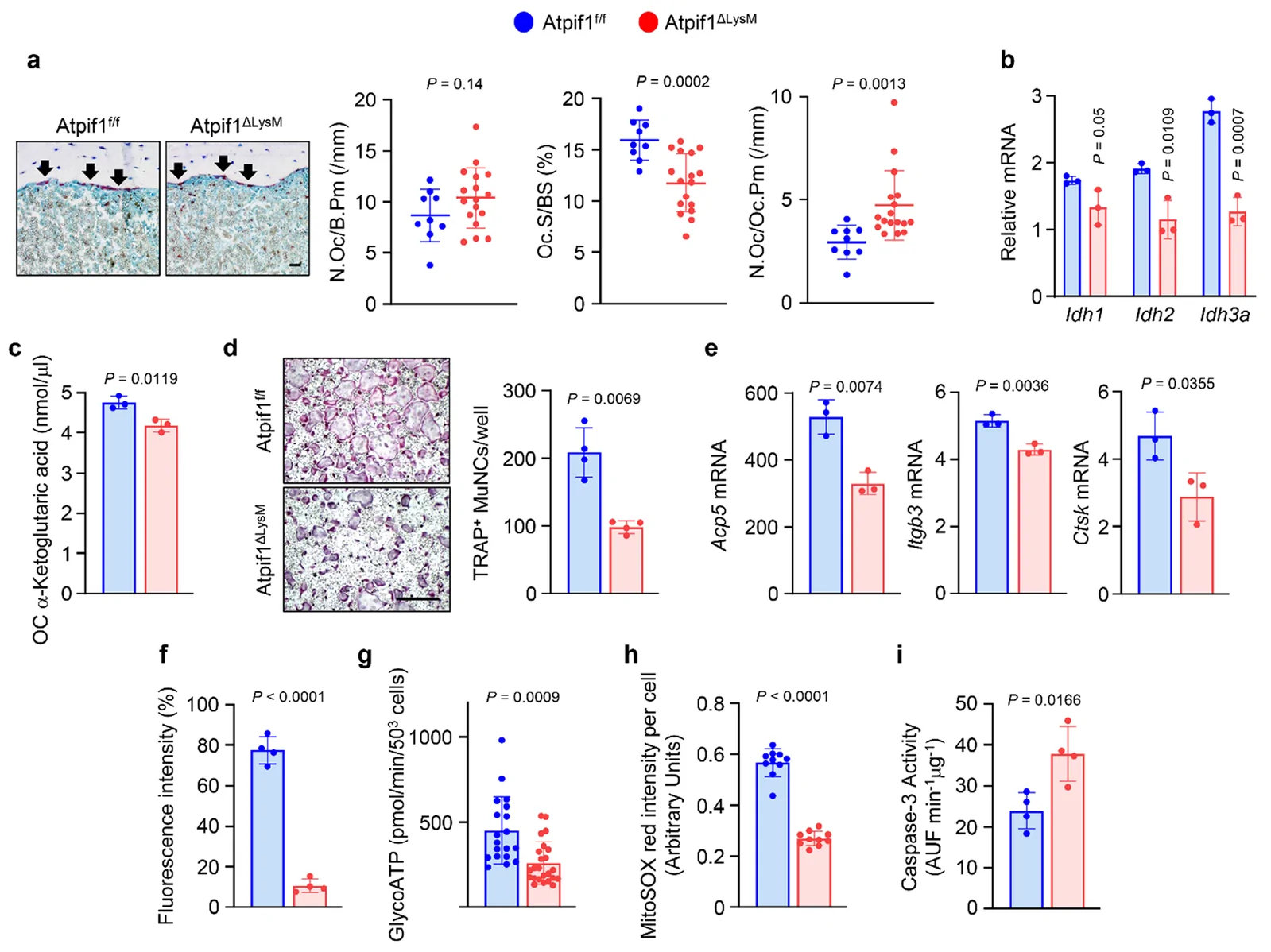
Deletion of *Atpif1* in the osteoclast lineage impairs osteoclast metabolic function. (**a**) Representative images used for osteoclast size assessment and histomorphometric analyses of the femoral endocortical surface in control and *Atpif1*^ΔLysM^ mice. Scale bars, 100 mm. (*left*) Number of osteoclasts per bone perimeter (N.Oc/B.Pm). (*middle*) Osteoclast surface per bone surface (Oc.S/BS). (*right*) Osteoclast number relative to osteoclast perimeter (N.Oc/Oc.Pm). (**b–i**) *Ex vivo* analyses of osteoclasts generated from aged control and *Atpif1*^ΔLysM^ mice. (**b**) Relative mRNA expression of Idh family members. (**c**) a-ketoglutarate levels in cultured osteoclasts. (**d**) Representative TRAP staining images and quantification of TRAP-positive multinucleated cells (MuNCs) per well. Scale bars, 500 mm. (**e**) Relative mRNA expression of osteoclast marker genes. (**f**) Quantification of resorption activity. (**g**) Glycolytic ATP production. (**h**) MitoSOX Red fluorescence intensity per cell. (**i**) Caspase-3 activity in osteoclasts generated from aged control and *Atpif1*^ΔLysM^ mice. Data are means ± s.d. Statistical analyses were performed using unpaired two-tailed Student’s t-test, repeated-measures ANOVA, or two-way ANOVA, as appropriate. Exact *P* values are shown in the panels. For *in vivo* skeletal phenotyping, n = 6–17 animals/group. All *ex vivo* measurements were performed in cultured BMMs pooled from 5 mice/group.

**Figure 7:**
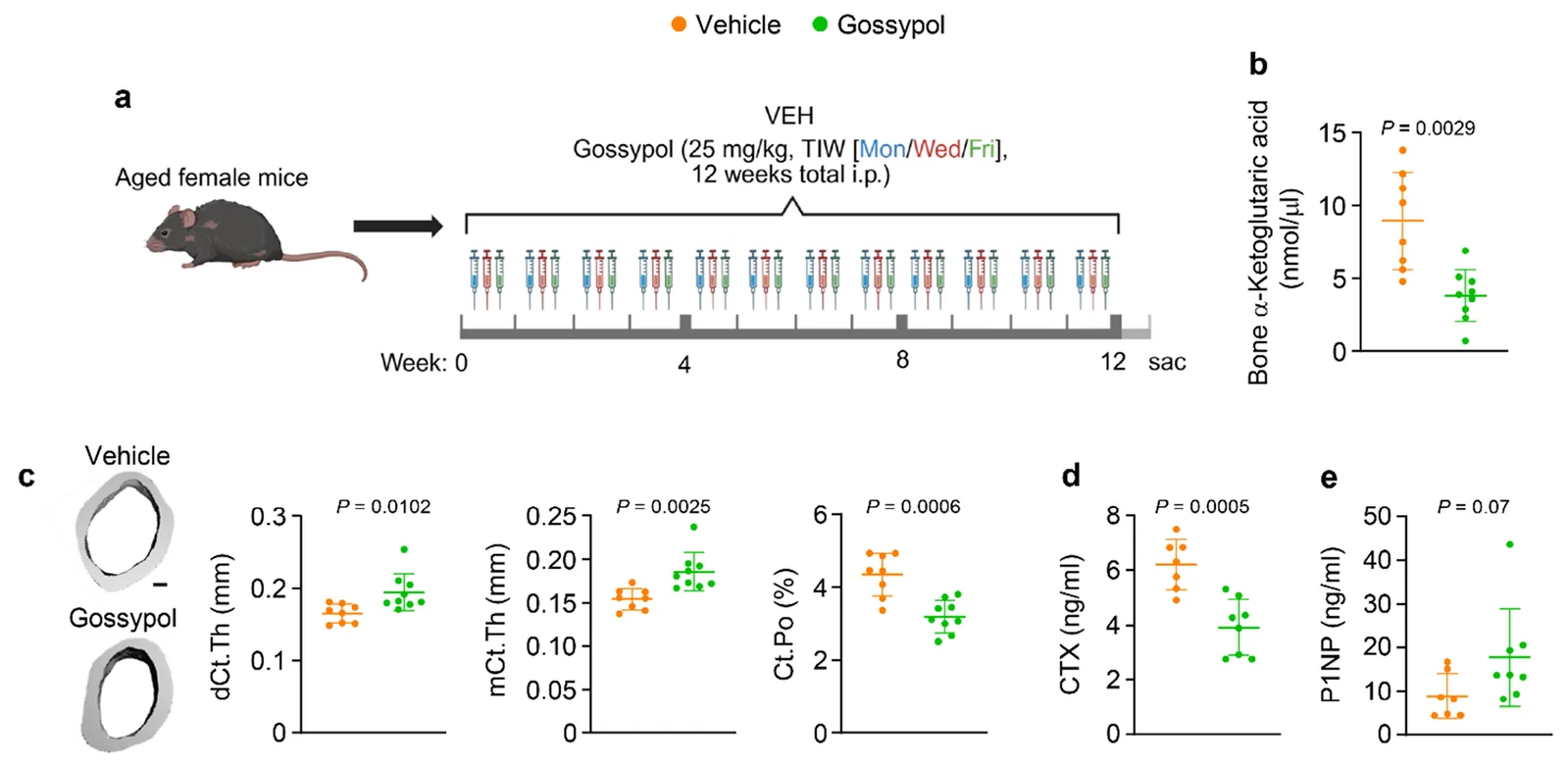
Gossypol attenuates age-related cortical bone loss in aged female mice. (**a**) Experimental scheme for gossypol treatment in aged female mice (n = 7–9 animals/group). (**b**) a-Ketoglutarate levels in bone shaft samples from vehicle- and gossypol-treated mice. (**c**) Representative cross-sectional micro-CT images of femoral midshaft cortical bone and micro-CT–based analysis of cortical bone parameters in vehicle- and gossypol-treated mice. Scale bars, 100 mm. (*left*) Cortical thickness at the femoral diaphysis (dCt.Th). (*middle*) Cortical thickness at the femoral metaphysis (mCt.Th). (*right*) Cortical porosity (Ct.Po). (**d**) Serum CTX and (**e**) P1NP levels in vehicle- and gossypol-treated mice. Data are means ± s.d. Statistical analyses were performed using an unpaired two-tailed Student’s t-test. Exact *P*values are shown in the panels.

## Data Availability

Processed metabolomics data, raw spectral data, and associated metadata generated in this study have been deposited in the NIH Common Fund’s National Metabolomics Data Repository (NMDR), Metabolomics Workbench, under Project ID PR003321 and Study ID ST005150 (Project DOI: 10.21228/M8DG4Z). Source data are provided with this paper, and values for all data points in graphs are reported in the Supporting Data Values file. All other data supporting the findings of this study are available from the corresponding author upon reasonable request.
